# Multi-voxel pattern analysis of face and word encoding fMRI in people with temporal lobe epilepsy and healthy individuals

**DOI:** 10.1016/j.yebeh.2025.110646

**Published:** 2025-09-06

**Authors:** Pegah Khosrapanah, John S. Duncan, Geoff J.M. Parker, Meneka Kaur Sidhu

**Affiliations:** a UCL Hawkes Institute, Department of Medical Physics and Biomedical Engineering, University College London, London, the United Kingdom of Great Britain and Northern Ireland; b Department of Clinical and Experimental Epilepsy, University College London, London the United Kingdom of Great Britain and Northern Ireland; c MRI Unit, Chalfont Centre for Epilepsy, Bucks, the United Kingdom of Great Britain and Northern Ireland; d Bioxydyn Limited, Manchester, the United Kingdom of Great Britain and Northern Ireland; e Oxford Brain Diagnostics, Oxford, the United Kingdom of Great Britain and Northern Ireland

**Keywords:** Epilepsy, Face and word encoding, Multivariate Pattern Analysis, Hippocampal sclerosis, Functional MRI

## Abstract

Memory functional MRI (fMRI) has been used to explore cognitive processing in people with refractory temporal lobe epilepsy (TLE) to predict memory decline after anterior temporal lobe resection (ATLR). Traditional studies employed univariate analysis (UVA), focusing on isolated voxel activity in mesial temporal regions. By contrast, multivariate pattern analysis (MVPA), examines distributed activity patterns, offering deeper insight into neural networks supporting cognitive functions.

MVPA allows precise mapping of individual memory encoding strategies that may influence surgical planning. Individual MVPA maps may be useful in the planning of surgical resection and trajectories for less-invasive procedures like laser interstitial thermotherapy (LiTT) to avoid critical brain regions. These may mitigate against the cognitive consequences of epilepsy surgery.

We used MVPA to examine face and word encoding differences in healthy individuals and in people with TLE due to hippocampal sclerosis (HS). In healthy controls, medial temporal regions bilaterally supported face and word encoding. Lateral temporal regions were lateralized—right for faces and left for words. The fusiform gyri were involved in both tasks across all participants. HS patients showed altered encoding patterns, with reduced classifier accuracy (CA) in medial and lateral temporal regions.

For face encoding, both left HS (LHS) and right HS (RHS) groups showed bilateral CA reductions in the parahippocampal gyrus (PHG), with additional hippocampal CA reduction in LHS. For word encoding, RHS patients showed CA reductions in right medial and bilateral lateral temporal regions, while LHS patients had reductions only in the left superior temporal gyrus (STG).

These results highlight the bilateral network involved in memory encoding and the value of individualized cognitive mapping to prevent surgery-associated memory decline.

## 1 Introduction

Episodic memory is the process of consolidating everyday experiences with their contextual features into a distinct neural trace. Critical processes include memory encoding, consolidation and memory retrieval. Memory encoding involves semantic and phonological elaboration, material organisation and manipulation before a distinct neural trace can be stored. Individual processes may differ according to the varied strategies employed to encode a particular neural trace [1,2].

Critical brain regions for encoding, as described in functional MRI (fMRI), computational modelling and lesional studies, include not just the hippocampi within the medial temporal lobe (MTL), but also the parahippocampal gyrus (PHG), fusiform gyrus (FG), lateral temporal gyri, posterior parietal lobe, frontal lobe (orbitofrontal, inferior and middle frontal gyrus) and subcortical structures [3,4,5,6].

The Material-Specific Memory Framework is a theory that suggests memory processes in the brain are organized based on the type or “material” of information being processed. According to this framework, verbal information activates left-sided networks during memory encoding and retrieval, while visual-spatial information recruits right-sided regions [7]. By contrast, the Task-Specific Memory model is a theoretical framework suggesting that the brain’s memory processes are organized based on the specific demands or requirements of a task rather than the type of material being processed, This model posits that different brain regions are recruited depending on the cognitive processes required and this perspective focuses on the functional demands of tasks, even if the material varies [8].

Hippocampal sclerosis (HS) is a common cause of drug-resistant temporal lobe epilepsy (TLE) that may be amenable to surgical resection. Material specific verbal and visual memory episodic memory impairment has been consistently described in left and right TLE respectively [9,10]. More recently however, more widespread cognitive deficits have been described in TLE that extend beyond this material specific framework ([11,12].

The concept of material specific strengths in a person’s memory profile is used to estimate how likely it is that an individual’s memory will decline after resective surgery (e.g. better pre-operative verbal memory may indicate a higher risk of verbal memory decline after left temporal lobe resection). Whilst verbal memory deficits have been seen in up to 40 % of individuals after language-dominant anterior temporal lobe resection, this has also been described after non-dominant resections [13]. This is in concert with connectivity studies that show a widespread network associated with memory processing [12].

Visual memory decline is seen after up to 20 % of non-dominant anterior temporal lobe resections. Temporal lobe lesionectomies where MTL structures have been spared have also been associated with deficits in immediate and delayed recall of verbal material [14,15], highlighting the critical role of lateral temporal neocortical structures in episodic memory processes. Neuroimaging studies indicate top-down input from middle and inferior temporal gyri for episodic verbal memory processing [16] and visual item representation [17]. Single neuron studies further detailed that there is also differential engagement of the lateral temporal cortex related to the timing of neural activity involved in early and late aspects of l memory encoding [18].

Whereas conventional univariate analyses (UVA) focus on individual voxel activity in isolation or averaged across all voxels in a region of interest (ROI), investigating memory representations using machine learning (multivariate pattern analysis, (MVPA)) capitalizes on the rich information represented in spatially distributed activity patterns with varying stimuli [19]. This enables a more comprehensive understanding of how distributed neural networks support cognitive processes [20]. A commonly used approach involves training a support vector machine (SVM) classifier to recognise the patterns of voxel-level activity linked to certain stimuli. The classifier is then applied to datasets that were not used for training. If the classifier identifies the pattern accurately, this suggests that the brain region holds a discernible trace of that individual stimulus. We described memory MVPA applied to the medial temporal lobe (MTL) structures in 10 people with TLE and HS [10,43]. This was an autobiographical memory retrieval rather than material specific encoding study. Participants were asked to recall specific scenes in the scanner. These were scenes ‘learnt’ in a forty-minute pre-scanning training session. The ROIs were limited to the hippocampus, para-hippocampal gyrus and entorhinal/perirhinal cortex. In this study, there were no discernible memory representations within the sclerotic hippocampus but the adjacent and contralateral MTL ROIs contained detectable information about the memories.

Minimally invasive techniques for medication-refractory focal epilepsy, such as laser interstitial thermal therapy (LiTT) [21], may have fewer consequences on cognition than ATLR. However, trajectories through, and the ablation of, critical brain regions may still result in less favourable cognitive outcomes.

Individual memory fMRI allows us to build a ‘road map’ when designing individual surgical implantations and resection strategies. MVPA in memory fMRI has several advantages over univariate approaches, primarily due to its ability to capture more complex patterns of activity that are often subtle and distributed across regions. UVA typically focuses on voxel-wise or ROI activations, comparing activity levels in each voxel independently. This approach may miss important information about how multiple brain areas interact or about low-level, fine-grained patterns across voxels and provides less information about the specific content or quality of the memory representations. Furthermore, MVPA uses cross-validation to confirm that the identified patterns generalize across different subjects or tasks. This helps to ensure that the patterns are consistent and not simply due to random noise. UVA does not inherently use cross-validation as part of the analysis, potentially making it less robust against inter-subject variability in memory processing.

We aimed to use MVPA to analyse pattern differences of face and word memory representations in people with left and right TLE and HS compared to one another and healthy controls. We tested the hypotheses that:

1) Memory MVPA will be useful in detecting patterns associated with face and word encoding processes in all participants.
2) Regions involved in encoding are likely to include MTL structures such as the PHG, FG (word and face areas), HC and lateral temporal neocortical gyri.
3) There will be reduced classifier accuracy in the ipsilesional MTL and neocortical structures in people with HS.

## 2 Methods

### 2.1 Subjects

41 Patients with TLE and unilateral HS who underwent preoperative evaluation at the National Hospital for Neurology and Neurosurgery in London were included in this study. 18 had left TLE (LTLE), median age 40 years, range 19–54 years and 23 right TLE (RTLE) median age 44 years, range 21–56 years. All patients underwent 3 T structural magnetic resonance imaging (MRI) which included measurements of hippocampal volumes and T2 relaxometry. These measurements confirmed the presence of unilateral HS and that the contralateral medial temporal regions were normal (Table 1). Seizure onset zones ipsilateral to the side of HS were confirmed through electroencephalography (EEG) video telemetry. All patients were native English speakers on anti-seizure medications. 22 healthy native English speakers without any history of neurological or psychiatric disorders were included in the evaluation

The median age of the healthy participants was 38 years, range19 to 58 years. Standardized neuropsychometry was administered to all individuals with TLE, and to healthy controls. Assessment of intellectual functioning was performed using Full-Scale IQ of the Wechsler Adult Intelligence Scale (WAIS) [22] in controls, and NART-IQ in people with TLE (reported to give the most reliable and precise estimates of patients’ Full-Scale IQ [23]. All participants’ verbal and visual memory was assessed using the verbal (VL) and design (DL) learning subtests of the BIRT Memory and Information Processing Battery version I (BMIPB-I) [24], as in previous focal epilepsy fMRI studies [12,13,25,26,27] (Table 1). Handedness and language dominance were determined with the standardized Oldfield questionnaire and language fMRI tasks. Controls and left/right HS groups were comparable for language dominance, handedness, binary sex, and age. Written informed consent was obtained from all participants in accordance with the Declaration of Helsinki. The NHNN and the UCL Queen Square Institute of Neurology Joint Research Ethics Committee approved this research. Exclusion criteria included contraindication to MRI, non-proficient English speaker, and intelligence quotient (IQ) < 70.

### 2.2 Magnetic resonance image acquisition

A 3 T General Electric Excite HDx MRI scanner with a gradient strength of 40 m/Tm and a slew rate of 150 Tm/s was used for the studies. For the functional MRI, gradient-echo echo-planar images were acquired, providing blood oxygen level-dependent (BOLD) contrast. Each volume comprised 36 contiguous oblique axial slices, slice thickness 2.5 mm (0.3 mm gap), field of view 24 cm x 24 cm, matrix 96 × 96 interpolated to 128 × 128 during image reconstruction, in-plane resolution 2.5, SENSE factor 2.5, echo time (TE) 25 ms, repetition time (TR) 2.75 s. The field of view was positioned to cover the temporal and frontal lobes with the slices aligned with the long axis of the hippocampus on the sagittal view. T1-weighted images were acquired using a coronal magnetization-prepared rapid gradient-echo (MP-RAGE) sequence with flip angle of 11°, field of view (FOV) of 256 mm × 256 mm × 170 mm, and a voxel size of 0.937 mm x 0.937 mm x 1.1 mm.

### 2.3 Memory encoding paradigm

During a single scanning session, participants were presented with the same non-famous faces and concrete nouns on a magnetic resonance compatible screen viewed through a mirror [10,13]. Visual and verbal items were presented for 3 s in blocks with a total of 10 blocks. One block contained 10 faces and 10 words followed by crosshair fixation. Jitter and random sampling were introduced through a 3 s interstimulus interval (vs. 2.75 s TR).

Participants were instructed to memorise information for future recall outside the scanner. With the aid of a joystick, participants completed a deep encoding task that required them to rate each stimulus as either pleasant or unpleasant. Face and word recognition were examined independently in an out-of-scanner recognition task forty minutes after scanning. The same 100 objects were mixed with an extra 50 new faces or words as foils in a random order for each recognition task, which was presented to subjects at the same pace as the scanner’s display of the items.

To indicate whether something was remembered, familiar, or novel, a button box was employed. Using these responses, each item displayed by the scanner was categorised as remembered, familiar, or forgotten. For both faces and words, Recognition Accuracy (RA) (%) was calculated (true positive and false positive).

### 2.4 Regions of interest (Anatomical mask for MVPA)

We specifically focused on HC, PHG, FG, and lateral temporal gyri comprising superior (STG), middle (MTG), and inferior temporal gyrus (ITG). In each hemisphere, automated ROI segmentation was performed using T1-weighted structural magnetic resonance imaging. An in-house script HIPPOSEG extraction tool was used for hippocampus segmentation [28,29]. We utilised FreeSurfer (v6.0) [30] for the remaining ROIs. Each segmentation was visually inspected and, if necessary, manually corrected. The MTL ROIs (HC, PHG, FG) and lateral temporal neocortical ROIs (STG, MTG, ITG) were grouped using MarsBaR ROI toolbox for SPM12 to study grouped medial and lateral temporal lobe contributions to memory encoding.

### 2.5 fMRI data pre-processing

Pre-processing of the fMRI data was performed using SPM12 (https://www.fil.ion.ucl.ac.uk/SPM). The fMRI images were realigned to correct for motion effects, slice-dependent delays adjusted, co-registered to individual’s native space MRI and minimally smoothed with a 2 mm FWHM Gaussian kernel. A high-pass filter was run on the images to remove signal changes due to scanner drift [31]. The data were then convolved with the canonical haemodynamic response function (HRF) [32]. Regressors of interest were generated by constructing three box-car functions for rest, faces, and words. A general linear model was created with one regressor for each trial and additional regressors of no interest, such as motion parameters. A set of beta maps that were each connected to a specific experimental trial were produced as a result of model parameter estimation. Beta values were then used to create the vectors used as inputs by the decoding algorithm. There were 10 blocks per participant, each containing 10 faces presented for 3 s each (30 s face block in total), 10 words for 3 s each (30 s word block) and rest (15 s rest block). 10 beta maps were obtained for each participant per type of trial (faces, words and rest).

### 2.6 MVPA processing

We implemented MPVA using Pattern Recognition for Neuroimaging Toolbox (PRoNTo), version 3 [33]. For each subject two classification tasks were considered: face encoding vs rest and word encoding vs rest. For each classification task a support vector machine was trained for each ROI. We trained the models and optimized their hyperparameters using a nested cross-validation approach for both classification tasks. The model’s performance (accuracy) was evaluated using the external loop, and the model’s hyperparameters (soft-margin parameter C) were optimized using accuracy as the optimization criterion in the inner loop. A “leave-one-subject-out” cross-validation scheme was employed for both the internal and external loops. The training set is further separated into training and testing sets for each outer loop fold based on the chosen cross-validation technique (leave-one-subject-out). According to our data, the outer loop was validated for 10 % of beta maps (1 beta map) and trained on a subset of 90 % of beta maps (9 beta maps). The inner loop is used to train and test the model with each value of the hyperparameter (default). The outer loop then uses the parameter that yielded the greatest performance in the inner/nested loop (based on Mean Squared Error for regression and balanced accuracy for classification). The model is evaluated on the excluded data (the data that were not utilized for parameter optimization) and trained using the “optimal” value of the hyperparameter for each fold of the outer loop [34]. To assess statistical significance of cross-validated accuracy of a model for each subject in each experiment and related ROI, we used non-parametric permutation tests with 500 permutations. The balanced accuracy for each subject which is the mean accuracy of each class over folds was used.

We examined the pattern of representation by dividing the temporal lobe into medial and lateral ROIs in each hemisphere for each group. The significance of classification accuracy (CA) was next examined within sub segments of the medial and lateral temporal segments in each hemisphere.

Although permutation tests provide the statistical significance of observed classification accuracy, comparing the observed classification accuracy to the level of chance determines if the classifier is performing above baseline. In neural decoding studies, the significant performance (classification accuracy) of a classifier is frequently evaluated by how closely it reaches the maximum correct classification rate of 100 % or, alternatively, by how far it deviates from chance-level depends on how many classes exist in data. For instance, all of the classification tasks in our analysis are two-class classification tasks with a conventional level of chance of 0.5. Although these probabilistic chance levels are frequently used in studies of brain signal categorization, they can present issues, as theoretically they are only valid for infinite sample sizes [35]. Therefore, in this study for each group we utilised the binomial cumulative distribution to derive statistical significance thresholds based on sample size. We used Kruskal-Wallis test followed by a Fisher’s least significant difference test for multiple comparisons based on the total number of ROIs. For grouped ROIs, multiple correction was based on four ROIs (left and right grouped medial and lateral ROIs). For sub-ROIs, correction was based on six total ROIs for mesial and neocortical regions separately.

We investigated CA within each group; controls, LHS and RHS and significant differences within ROIs between the groups.

## 3 Results

### 3.1 Neuropsychological performance and clinical parameters

Both individuals with LHS and with RHS had lower IQs and performed significantly worse than controls on the face and word recognition tasks as well as list and design learning (2-tailed *t*-test p < 0.05; Table 1). Individuals with LHS and RHS did not differ significantly in age, age at the onset of epilepsy, epilepsy duration, IQs or list learning, face and word recognition (2-tailed *t*-test p > 0.05, Table 1). People with RHS had significantly lower performance on design learning than people with LHS.

**Fig. 1:**
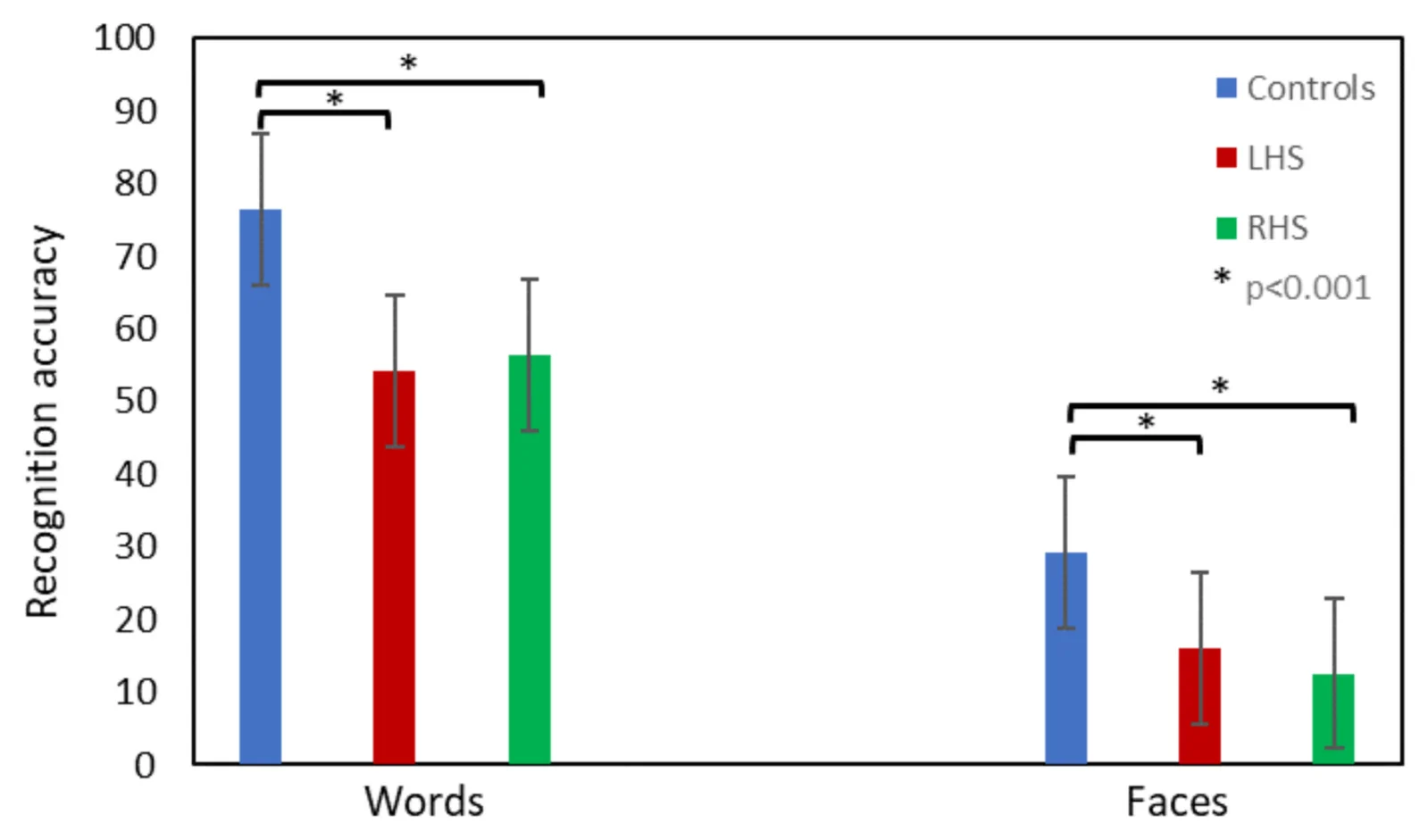
Histograms showing mean percentage behavioural in-scanner task recognition accuracy with error bars (± 1 SD) for word and face recognition across all groups. LHS = left hippocampal sclerosis; RHS = right hippocampal sclerosis.

Control subjects demonstrated significantly higher performance than people with LHS and people with RHS in both word and face recognition (Table 1). There was no significant difference observed in word and face recognition accuracy between the LHS and RHS groups. Both control subjects and people with HS showed significantly higher accuracy in recognizing words than in recognizing faces (Table 1 and Fig. 1).

### 3.2 Level of chance Calculations

The threshold for determining chance from statically significant difference within each group CA was determined using binomial cumulative distribution. The calculated chance level was 68 % in controls, 70 % in LHS and 65 % in RHS. After correction for multiple comparisons, the CA threshold level of chance was 73 % in controls, 75 % in LHS and 74 % in RHS in grouped ROIs, and 77 % for sub-ROIs in controls. Sub-ROI CA level of chance was identical to grouped ROI CA in both LHS and RHS.

### 3.3 Group analysis: Faces vs rest

All p-values are displayed in supplementary table S1.

### 3.4 Controls

In controls, significant CA was seen in both the grouped left and right medial temporal ROIs and the right grouped lateral temporal ROI. Considering individual ROIs, above chance classification was seen in the PHG, FG, superior, middle and inferior temporal gyri bilaterally.

**Fig. 2:**
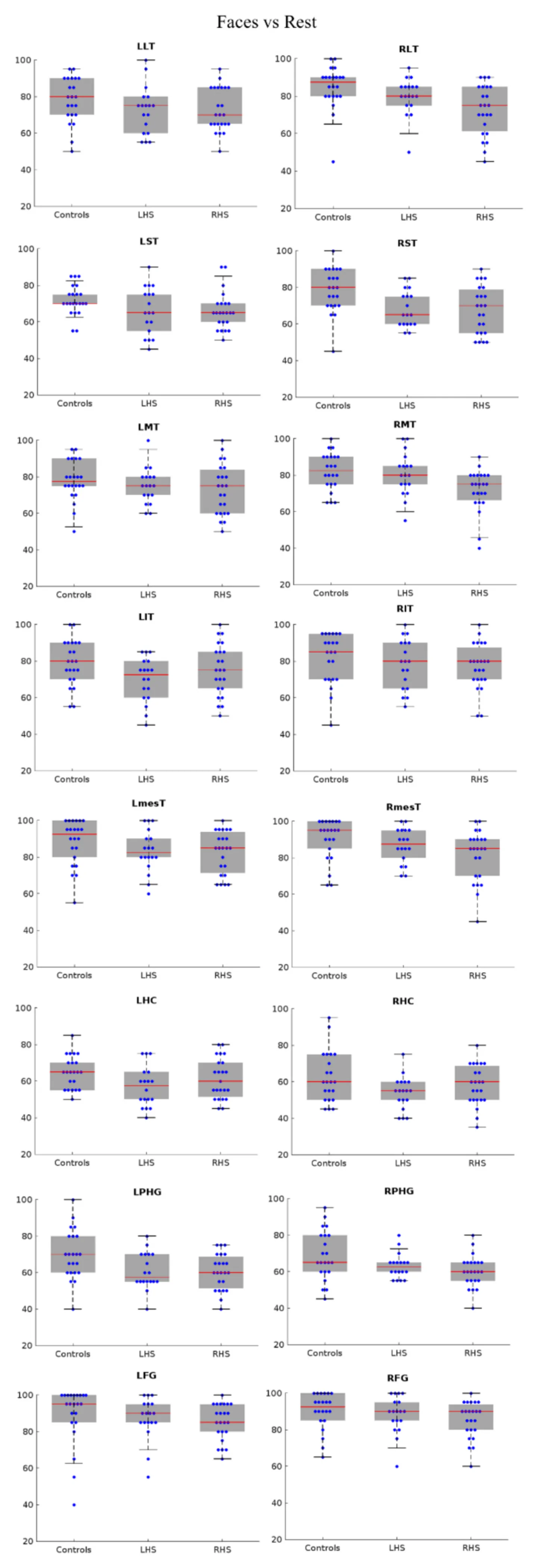
Box plots showing classification accuracy distributions within the grouped lateral and medial ROIs (first row) and the 12 sub-ROIs within the left and right hemispheres in all three groups for face encoding vs rest. Each plot shows the median, interquartile range, the lower/upper adjacent values and kernel density of the classifier performance for each group. Significant group difference was noted in the grouped right lateral ROI and right superior and middle temporal sub-ROIs. LHS: Left Hippocampal Sclerosis, RHS: Right Hippocampal Sclerosis L: left, R: right, LT: lateral Temporal grouped, mesT: mesial temporal grouped, ST: Superior Temporal, HC: Hippocampus, MT: Middle Temporal, PHG: Parahippocampal Gyrus, IT: Inferior Temporal, FG: Fusiform Gyrus.

Significant CA after correction for multiple comparisons was seen in the fusiform, inferior and middle temporal gyri bilaterally and in the right superior temporal gyrus (Table 2). This is also presented as a box plot (Fig. 2) and in Fig. 4.

### 3.5 LHS

Significant CA above chance was detected in the left and right medial and lateral temporal grouped ROIs. Within the subregions, there was significant CA within the right inferior temporal and bilateral middle temporal and fusiform ROIs.

After correction for multiple comparisons, the left and right medial and right lateral temporal regions demonstrated significant CA. Within the subregions, right middle and inferior temporal and bilateral fusiform ROIs showed significant CA above the level of chance (Table 2). This is also presented as a box plot (Fig. 2) and in Fig. 4.

### 3.6 RHS

Significant CA above chance was detected in the left and right medial and lateral temporal grouped ROIs. Within the subregions, there was significant CA within the superior, middle and inferior temporal gyri and fusiform gyri bilaterally.

After correction for multiple comparisons, the right inferior temporal gyrus and bilateral fusiform regions showed above chance CA. (Table 2). This is also presented as a box plot (Fig. 2) and in Fig. 4.

Average CA over all folds of each individual in each ROI has been reported for word encoding task in the supplementary material (Table S3).

### 3.7 Significant group differences (considering all 3 groups)

After multiple corrections using Kruskal-Wallis test followed by a Fisher’s least significant test, the main differences in CA among the three groups were noted in the right lateral grouped ROIs, primarily the right superior and middle temporal sub-ROIs (p < 0.008).

### 3.8 Controls vs LHS

Before multiple correction, people with LHS showed lower CA within the HC, PHG and superior temporal ROIs bilaterally and predominantly left middle and inferior temporal ROIs compared to controls. Following multiple corrections, individuals with LHS showed significantly lower CA within the right superior temporal ROI compared to controls.

### 3.9 Controls vs RHS

Individuals with RHS showed lower CA within the right medial and lateral grouped ROIs than controls. Within the sub ROIs, people with RHS showed reduced CA within the right middle temporal, left fusiform and superior temporal ROI and PHG bilaterally. After correction for multiple comparisons individuals with RHS showed significantly lower CA within the right medial and lateral grouped ROIs and right middle temporal sub-ROI compared to controls.

### 3.10 LHS vs RHS

There was higher CA in the right middle temporal gyrus in people with LHS than in people with RHS. Following multiple correction there were no significant differences in all regions between the LHS and RHS groups.

### 3.11 Group analysis: Words vs rest

All p-values are displayed in supplementary table S2.

### 3.12 Controls

In controls, significant CA was seen in the grouped medial and lateral temporal ROIs, bilaterally. Considering individual ROIs above chance classification was seen in the FG and inferior temporal gyri bilaterally and left middle and superior temporal gyri.

**Fig. 3:**
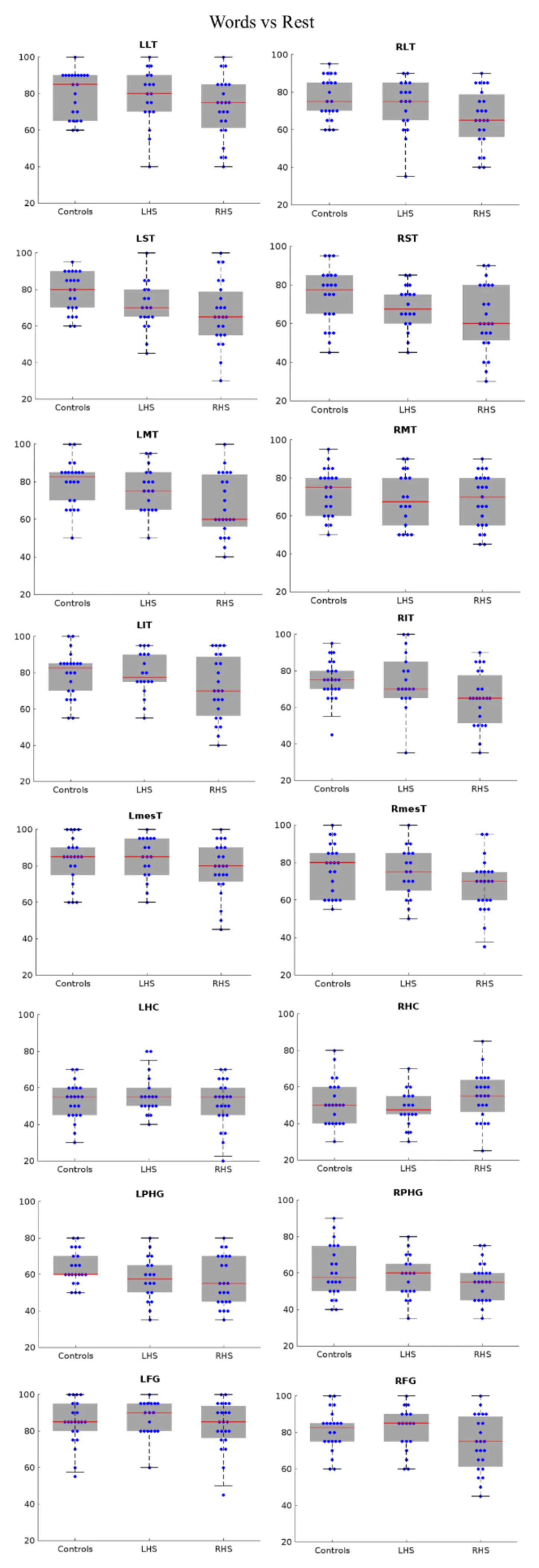
Box plots showing classification accuracies within the four lateral and medial grouped ROIs (first row) and the 12 sub-ROIs within the left and right hemispheres in all three groups for the word encoding vs rest scenario. Each plot shows the median, interquartile range, the lower/upper adjacent values and kernel density of the classifier performance for each group. Significant group difference in CA was noted in the left medial temporal gyrus. LHS: Left Hippocampal Sclerosis, RHS: Right Hippocampal Sclerosis L: left, R: right, LT: lateral temporal grouped, mesT: medial temporal grouped, ST: superior temporal, HC: hippocampus, MT: middle temporal, PH: parahippocampal gyrus, IT: inferior temporal, FG: fusiform gyrus.

**Fig. 4:**
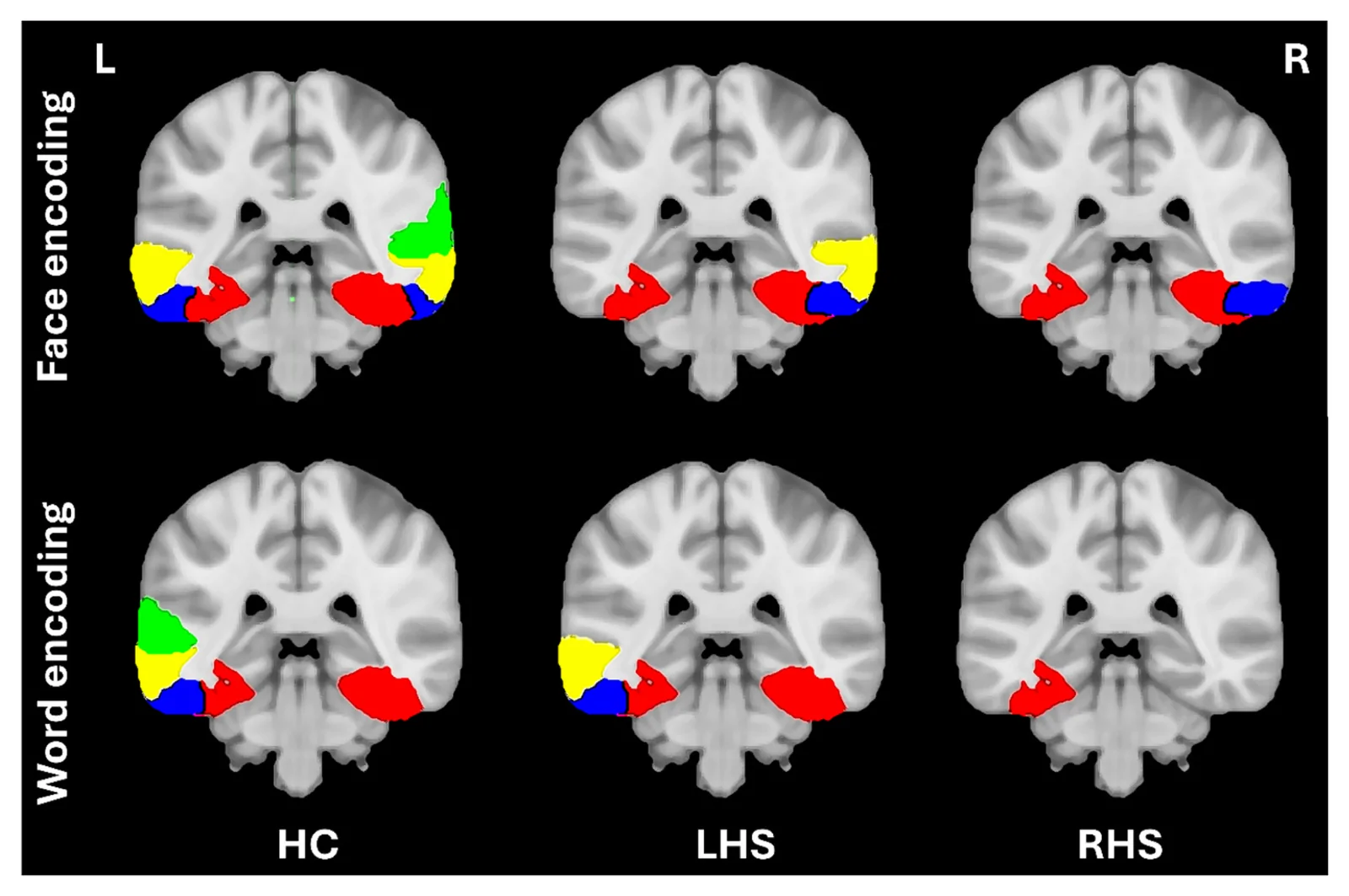
Brain regions with significant CA after multiple correction in different groups (HC, LHS and RHS) in face encoding (top row) and word encoding (bottom row) in colour coded ROIs; Fusiform gyrus (red), inferior temporal gyrus (blue), middle temporal gyrus (yellow) and superior temporal gyrus (green). HC: Healthy Controls, LHS: Left Hippocampal sclerosis, RHS: Right Hippocampal sclerosis. (For interpretation of the references to colour in this figure legend, the reader is referred to the web version of this article.)

After multiple comparisons, the left medial and lateral temporal grouped ROIs (p < 0.0125) and left superior, middle and inferior temporal and bilateral fusiform sub-ROIs, revealed significant CA above the level of chance (p < 0.008) (Table 3). This is also presented as a box plot (Fig. 3) and in Fig. 4.

### 3.13 LHS

Significant classification accuracy over chance was detected in the medial and lateral grouped temporal ROIs bilaterally. Considering individual ROIs above chance classification was seen in FG bilaterally and left middle, and inferior temporal gyrus within grouped ROIs. After multiple comparisons corrections, the left medial and lateral temporal grouped ROI, middle and inferior temporal and bilateral fusiform ROIs showed significantly higher classification accuracy above the level of chance (Table 3). This is also presented as a box plot (Fig. 3) and in Fig. 4.

### 3.14 RHS

Significant classification accuracy over chance was detected in the left medial grouped temporal ROIs. Considering individual ROIs above chance classification was seen in the left FG. Similar to controls, following multiple comparisons correction, the left medial temporal grouped ROI and the left fusiform ROI within it showed significant CA above the level of chance (Table 3). This is also presented as a box plot (Fig. 3) and in Fig. 4.

Average CA over all folds of each individual in each ROI has been reported for face encoding task in the supplementary material (Table S4). Fig. 3 represents box plots of average classification accuracies in different ROIs of each group in the words vs rest encoding experiment (Controls, LHS and RHS).

### 3.15 Significant group differences (considering all 3 groups)

After multiple corrections using Kruskal-Wallis test followed by a Fisher’s least significant test, the main difference in CA among the three groups was noted in the left middle temporal gyrus.

### 3.16 Controls vs LHS

For words vs rest, the left superior temporal ROI showed significantly higher CA in controls than in individuals with LHS. After correction for multiple comparisons, none of the ROIs had significant differences in CA.

### 3.17 Controls vs RHS

People with RHS showed lower CA in the right medial and lateral grouped ROIs. Within the sub-ROIs people there was reduced CA within the right superior and inferior temporal ROIs, and left PHG, superior and middle temporal ROIs in RHS compared to controls. Following multiple correction the left middle temporal ROI showed significantly lower CA in people with RHS compared to controls.

### 3.18 LHS vs RHS

Individuals with LHS showed significantly higher CA in the right ITG and left medial temporal grouped ROI than people with RHS. After multiple correction left medial temporal grouped showed significantly higher CA in LHS.

All ROIs with significant classification accuracies after correction for multiple comparisons in controls, LHS and RHS for faces vs rest and words vs rest are summarised in Table 4 and Fig. 4.

## 4 Discussion

Multivariate pattern analysis showed that word and face encoding are associated with distinct representations in healthy controls and people with epilepsy. In healthy controls, this is bilateral within the medial temporal grouped ROIs for face and word encoding but left lateralised in the lateral temporal ROIs for words and right lateralised in the lateral temporal ROIs for faces. Within the medial temporal lobe, the FG plays a critical role for both word and face encoding in controls and patients alike. Only controls showed above chance CA in the PHG for faces (chance being 68 % in controls).

People with both LHS and RHS show varied patterns of face and word representations in both hemispheres compared to controls suggesting impacts beyond material specific effects that have been traditionally accepted.

For face encoding, there were significant reductions in bilateral PHG CA compared to controls in both left and right HS and bilateral reduction in HC CA in people with LHS. Neocortical reductions were primarily right sided in RHS. This implies widespread but most significantly ipsilesional face encoding pattern recognition disruption in people with RHS.

For word encoding, RHS showed right medial temporal CA reductions compared to controls. However, CA was reduced bilaterally over the lateral temporal ROIs. In LHS, CA was reduced only in the left superior temporal ROI, indicating intact word pattern recognition function in the other left temporal neocortical regions; regions that are partly resected as part of a standard anterior temporal lobe resection. There was higher CA in the left grouped MTL in LHS compared to RHS but this was driven by superior CA in the left FG. This may represent greater reorganisation to more posterior MTL structures ipsilaterally in people with LHS.

Boxplots (Fig. 2 and Fig. 3) show the individual variability of pattern recognition in healthy controls and people with epilepsy In people with epilepsy, despite a seemingly homogenous pathology of HS, this suggests differing individual reorganisation patterns related to the underlying disease and possibly also varied mechanisms and strategies employed by individuals for these cognitive tasks. MVPA allows the investigation of spatial representations associated with individual neural traces of these individual processes.

### 4.1 Face Encoding

The neural basis of face perception has been studied extensively and provides insights into the computational and functional architecture of visual recognition and the brain [36,37]. Information-based brain mapping and dynamic discrimination analysis suggest that spatiotemporal patterns that support face classification at the individual level are supported by a bilateral fusiform and anterior temporal network [38]. Furthermore, the fusiform face area responds with distinct patterns of activation to different face identities [38]. Similarly, Rivolta et al demonstrated neural activity associated with faces lie within the ‘core’ areas that include the fusiform face area and face regions within the anterior temporal cortex bilaterally (D. [39].

Similarly, in controls we showed bilateral pattern representation within the mesial and lateral grouped temporal ROIs on face encoding, with the greatest pattern representation within the fusiform and temporal neocortical regions.

TLE is increasingly conceptualized as a network disorder [40] and correspondingly, in those with TLE, there is disruption in functional connectivity within both temporal and extratemporal regions [12,41]. In people with left and right HS, we showed bilateral medial and lateral temporal reduced MVPA representations within the HC, PHG and temporal neocortical regions. In our study both people with LHS and RHS had worse face and word recognition accuracy and design and list learning scores than controls. This is in keeping with a more task specific [8] rather than material specific model of cognitive difficulties such as memory, language, attention, executive, and social cognition difficulties in those with TLE.

In a different disease model, Rivolta et al. showed that in people with prosopagnosia there was a reduced ability to differentiate patterns associated with faces and objects within the occipital, fusiform face area, parahippocampal cortex and anterior temporal neocortex suggesting a common pathway disruption of facial pattern recognition regardless of the pathology [42].

We used box plots to demonstrate the more widespread distribution of classification accuracy amongst people with LHS and RHS than seen in in controls. The benefit of MVPA is the ability to show individual spatial representations associated with individual neural traces that may aid individual surgical planning for both dominant and non-dominant surgical resections.

Using a scene encoding task that was limited to the mesial temporal lobes, reduced classifier accuracy was seen in the ipsilesional hippocampi in people with TLE due to HS [43]. In this study, a non-material specific task which incurs bilateral rather than unilateral activations within the MTL, limits the ability to investigate or comment on contralateral reorganisation of unilateral processes [44].

Univariate face and word encoding connectivity studies highlighted the network related to efficient connectivity between medial temporal regions including the fusiform gyrus to temporal neocortical and extra-temporal regions in people with TLE [12]. Structural and functional connectivity studies that have showed an increased disruption of networks in TLE due to HS compared to other pathologies [45,46]. Correspondingly, people with HS had reduced connectivity to extra-temporal regions such as the insula but increased mesial temporal connectivity between the hippocampi and posterior fusiform gyrus in a face encoding study [12].

Using complementary MVPA analyses we showed that patterns for faces and words are significantly associated with representations in the FG. Future analyses will investigate anterior to posterior gradients of face and word representations within the MTL.

### 4.2 Word Encoding

Anterior temporal lobe resection in which the hippocampus is removed to the level of the tectal plate has traditionally been associated with up to a 40 % risk of verbal memory decline [47]. However, memory encoding and retrieval difficulties have also been critically affected in people who have had temporal neocortical lesionectomy with mesial temporal structures being undamaged [48,49]. In these procedures however, disconnection of critical white matter tracts may adversely affect the memory network. This particularly affects verbal and visual list learning and therefore the overall ability to successfully encode an item [49,50]. The ventral anterior temporal lobe plays a central role in semantic representation of both verbal and visual material [51,52]. A combined computational modelling utilising MVPA and electrocorticography (ECoG) showed that the anterior temporal lobe encodes distributed semantic representations that change dynamically and nonlinearly with stimulus processing, highlighting the role of this region as a key hub within the cortical semantic network [53].

Univariate and multivariate studies have indicated a top-down input from middle and inferior temporal gyrus for semantic and visual items representation with the MTG being particularly implicated in tasks related to lexical comprehension and semantic cognition [54]. This region is also vital for understanding visual messages and is thought to utilize contextual knowledge to retrieve the relevant semantic information required for these tasks [55,56].

Previous MVPA studies have also suggested a perceptual to conceptual gradient of semantic coding whereby real object size implied by a word appears to be primarily encoded in early visual regions, while the taxonomic category cluster is in more anterior temporal regions. This anteroposterior gradient of information content indicates that different areas along the ventral stream encode complementary dimensions of the semantic space [57]. We performed a word encoding task using neutral words, however strategies used to actively encode may vary from person to person. People who are more reliant on visual imagery may encode more posteriorly along the ventral stream, highlighting the role of individual MVPA when mapping eloquent areas preoperatively.

The role of the fusiform gyrus in word and face recognition has been well established with bilateral hemispheric contributions dependent on processing styles and levels of processing [58]. Furthermore, visual processing of words and word-like series of letters showed two discrete portions of the fusiform gyrus responded preferentially to letter-strings. A region of the posterior fusiform gyrus responded equally to words and non-words, and was unaffected by the semantic context in which words were presented. In contrast, a region of the anterior fusiform gyrus was sensitive to these stimulus dimensions [59] further differentiating the complex processing related to face and word recognition mechanisms and strategies employed.

In line with previous studies, we showed that healthy controls had significant representations for word encoding in the fusiform gyri bilaterally. However, lateral temporal representations were more significant in the left neocortical ROIs, displaying hemispheric lateralisation for words. People with LHS showed reduced representations within the left STG compared to healthy controls. People with RHS showed reduced representations within both the medial and lateral ROIs bilaterally. Interestingly, RHS showed lower representations in the left medial temporal ROI compared to LHS. This implies that people with RHS have more widespread word pattern disruption compared to those with LHS. This may contribute to the verbal memory difficulties noted in people with RHS in our study.

### 4.3 Strengths and Limitations

In this study, crucially, we used simple face and word tasks that have successfully been used to investigate the episodic memory network in people with epilepsy who have significant cognitive difficulties [9,10,12,60].

We applied stringent statistical techniques to describe the effects reported. Level of chance was not just determined by number of classification classes (in this case 50 % for two classes). We used binomial cumulative distributions to determine level of chance in each group. Given the large number of features typically involved in MVPA, we applied multiple comparison correction to control for false positives.

One of the strengths in this study is the relatively large number of participants with TLE due to HS (41)—especially given that some studies report group effects with as few as 9 participants. The limitation of this however is that we were unable to investigate differential patterns associated with TLE due to other pathologies.

Although both left and right TLE groups were matched for number of anti-seizure medications (ASM) the effect of different ASMs were not studied individually due to the number of different medications prescribed.

### 4.4 Clinical Implications

Traditionally, in pre-surgical multidisciplinary meetings, the concept of material specific strengths in a person’s memory profile is used to approximate the risk of memory decline with resective surgery. More recent studies have highlighted more widespread deficits in cognitive functions beyond the surgically resected area, exemplifying the network basis of cognitive functions that are more extensive and bilateral than previously appreciated [12].

Using univariate analyses, lateralisation of verbal memory activation within a fronto-temporal was the strongest predictor of verbal memory decline compared to standard clinical measures [26]. This method however does not take into account the specificity of regional representations of strategies utilised for encoding processes.

We showed the varied patterns associated with face and word encoding and how this can differ between patient groups and across individuals. Taking individual representations at the presurgical level into account can inform individual surgical strategies. These may suggest regions to spare not just when planning ablation or resection but also to plan trajectories to avoid critical regions to mitigate against cognitive decline following neurosurgical intervention.

To this effect, we are undertaking a further study to determine whether individual MVPA maps showing recognition accuracies within regions of interest are predictive of decline of verbal and visual memory function after temporal lobe resection.

## Supplementary Material

**Appendix A.Supplementary data**

Supplementary data to this article can be found online at https://doi.org/10.1016/j.yebeh.2025.110646.

Supplementary material

## Figures and Tables

**Table 1:** Demographic details in healthy controls and patients.

	Age (years/median)	Age at onset (years/median)	Duration (years/median)	Right HC Volume (cm^3^)	Left HC Volume (cm^3^)	Seizure frequency (average per month)	List learning/ 75 (median)	Design learning/45 (Median)	IQ	RA Words (average) %	RA Faces (average) %
**22 Controls (13 female)**	38	NA	NA	2.7	2.66	NA	56.5	41.5	113.5	76.4	29.2
**18 LHS (12 female)**	42	13.5	18.5	2.6	1.74*^&^	8.2 (range 2–240)	47.5*^&^	37.5*	99.5*	53.2*	16.4*
**23 RHS (19 female)**	44	14	24.5	1.86*^$^	2.69	7.64 (range 0.3–150)	43*	29*^$^	103*	56.4*	12.6*

* Significantly lower than control average (p < 0.05).

$ RHS significantly lower than LHS (p < 0.05).

& LHS significantly lower than RHS (p < 0.05). RA = Recognition Accuracy of in scanner task, HC=Hippocampus, LHS = Left Hippocampal Sclerosis, RHS = Right Hippocampal Sclerosis, IQ = Intelligence Quotient.

**Table 2:** Average classification accuracies in different ROIs in Controls, LHS and RHS for faces vs rest.

		Average classification accuracy (%)- CONTROLS			LHS			RHS		
		Left	Right		Left	Right		Left	Right	
Grouped ROI	MesT	88*	90*		83*	86*		82*	81*	
Sub-ROI	HC	65	62		57	52		61	59	
	PHG	69	68		60	62		62	61	
	FG	89*	90*		87*	90*		84*	84*	
Grouped ROI	Lateral temporal	78	84*		71	80*		72	72	
Sub-ROI	STG	71	78*		65	68		66	67	
	MTG	77*	82*		74	80*		71	72	
	ITG	79*	81*		70	79*		73	74*	

LHS: Left Hippocampal Sclerosis, RHS: Right Hippocampal Sclerosis, MesT: Medial Temporal, HC: Hippocampus, PHG: Parahippocampal gyrus, FG: Fusiform Gyrus, STG: Superior Temporal Gyrus, MTG: Middle Temporal Gyrus, ITG: Inferior Temporal Gyrus.

* Significant classification accuracy after correction for multiple comparisons.

**Table 3:** Average classification accuracies in different ROIs and their significance after correction for multiple comparisons in controls, LHS and RHS for words vs rest.

		Average classification accuracy (%)-CONTROLS			LHS			RHS		
		Left	Right		Left	Right		Left	Right	
Grouped ROI	MesT	83*	76*		83*	75		78*	68	
Sub-ROI	HC	52	51		56	49		51	54	
	PHG	63	61		57.5	58		56	55	
	FG	84*	80*		86*	81*		83*	73	
Grouped ROI	Lateral temporal	79*	76*		78*	73		71	66	
Sub-ROI	STG	78*	73		70	68		66	62	
	MTG	79*	72		76*	68		67	68	
	ITG	79*	75		78*	75		71	64	

MesT: Medial Temporal, HC: Hippocampus, PHG: Parahippocampal gyrus, FG: Fusiform Gyrus, STG: Superior Temporal Gyrus, MTG: Middle Temporal Gyrus, ITG: Inferior Temporal Gyrus.

* Significant classification accuracy after correction for multiple comparisons.

**Table 4:** Summary of ROIs with significant classification accuracies after correction for multiple comparisons in controls, LHS and RHS for faces vs rest and words vs rest.

	FACES VS REST					WORD VS REST			
		CONTROLS	LHS	RHS		CONTROLS	LHS	RHS	
	**Grouped Mes T**	Bilateral	Bilateral	Bilateral		Bilateral	Left	Left	
	**FG**	Bilateral	Bilateral	Bilateral		Bilateral	Bilateral	Left	
	**Grouped Lateral Temporal**	Right	Right			Bilateral	Left		
	**STG**	Right				Left			
	**MTG**	Bilateral	Right			Left	Left		
	**ITG**	Bilateral	Right			Left	Left		

LHS: Left Hippocampal sclerosis, RHS: Right Hippocampal sclerosis, Mes T: medial temporal grouped, FG: fusiform gyrus, STG: superior temporal gyrus, MTG: middle temporal gyrus, ITG: inferior temporal gyrus.
