# Supplementary material for: Multi-voxel pattern analysis of face and word encoding fMRI in people with temporal lobe epilepsy and healthy individuals
Source: Epilepsy Behav. Author manuscript; Available in PMC 2026 Sep 11. (PMC7619483; doi:10.1016/j.yebeh.2025.110646)
Supplement: Supplementary file 1 [file EMS217849-supplement-Supplementary_material.docx]

**Supplementary material:**

Table S1: Average classification accuracies in different ROIs and their significance values after correction for multiple comparisons in controls, LHS and RHS for faces vs rest.

|  |  | Average classification accuracy (%)-controls | | LHS | | RHS | |
| --- | --- | --- | --- | --- | --- | --- | --- |
|  |  | Left | Right | Left | Right | Left | Right |
| Grouped ROI | MesT | 88^*^ (9.7E-08) | 90^*^( 2.6E-08) | 83^*^(3.3E-06 ) | 86^*^(2.6E-08) | 82^*^(1.3E-07 ) | 81*(8.1E-06) |
| Sub-ROI | HC | 65 (0.06) | 62 (0.08) | 57(0.1) | 52(0.07) | 61(0.06) | 59 (0.1) |
|  | PHG | 69 (0.02) | 68 (0.03) | 60 (0.02) | 62(0.04) | 62 (0.09) | 61 (0.07) |
|  | FG | 89^*^(2 E-06 ) | 90^*^( 5 E-10) | 87^*^(2.9 E-07) | 90^*^(2.5 E-08) | 84^*^(9.4 E-10) | 84^*^(1.1E-09) |
| Grouped ROI | Lateral temporal | 78 (0.06) | 84^*^(5.9E-07) | 71(0.02) | 80^*^(8 E-06) | 72 (0.02) | 72 (0.04) |
| Sub-ROI | ST | 71 (0.02) | 78^*^(0.0002) | 65 (0.4) | 68(0.1) | 66 (0.03) | 67 (0.01) |
|  | MT | 77^*^(0.0002) | 82^*^(1.05E-06) | 74(0.01) | 80^*^(3.2 E-05) | 71(0.009) | 72 (0.009) |
|  | IT | 79*(0.0002) | 81*(7.6 E-05) | 69(0.1) | 79^*^(0.0006) | 73 (0.01) | 74*(8.4E-05) |
| MesT: Medial Temporal, HC: Hippocampus, PHG: Parahippocampal gyrus, FG: Fusiform Gyrus, ST: Superior Temporal, MT: Middle Temporal, IT: Inferior Temporal | | | | | | | |
| ^*^ Significant classification accuracy after correction for multiple comparisons | | | | | | | |

Table S2: Average classification accuracies in different ROIs and their significance values after multiple comparisons correction in Controls in the words vs rest experiment.

|  |  | Average classification accuracy (%) controls | | LHS | | RHS | |
| --- | --- | --- | --- | --- | --- | --- | --- |
|  |  | Left | Right | Left | Right | Left | Right |
| Main ROI | MesT | 83^*^(  1.3E-05) | 76*(  0.003  ) | 83^*^(  1.9E-06) | 75(  0.04) | 78^*^(  0.0001) | 68(  0.1) |
| Sub-ROI | HC | 52 (0.06) | 51(0.08) | 56(  0.07) | 49(0.2) | 51(0.1  ) | 54(0.07) |
|  | PHG | 63(  0.07) | 61(  0.08) | 57.5(  0.08) | 58(  0.08) | 56(  0.2) | 55(  0.1) |
|  | FG | 84^*^(  1.3E-06) | 80*(  1.4E-05) | 86^*^(  2.8E-08) | 81^*^(  2.1E-05) | 83^*^(  6.8E-07) | 73 (  0.06) |
| Main ROI | Lateral temporal | 79*(  0.0001) | 76*(  0.0008) | 78*(  0.002) | 73 (  0.02) | 71(  0.3) | 66(  0.3  ) |
| Sub-ROI | ST | 78*(  0.0001) | 73(  0.08) | 70(  0.09) | 68(  0.2) | 66(  0.2) | 62(0.2) |
|  | MT | 79*(  0.0001) | 72(  0.06) | 76*(  0.001) | 68 (  0.2) | 67(0.2) | 68(0.1) |
|  | IT | 79^*^(  0.0002) | 75(  0.04) | 78^*^(  0.0001) | 75 (  0.1) | 71(  0.08) | 64 (  0.4) |
| MesT: Medial Temporal, HC: Hippocampus, PHG: Parahippocampal gyrus, FG: Fusiform Gyrus, ST: Superior Temporal, MT: Middle Temporal, IT: Inferior Temporal Superior Temporal, MT: Middle Temporal, IT: Inferior Temporal  * Significant classification accuracy after correction for multiple comparisons | | | | | | | |

**Table S3:** Average classification accuracies of all folds in different ROIs in Controls, LHS and RHS individuals in the faces vs rest experiment**.**

| **Subjects** | **LmesT** | **LLT** | **RmesT** | **RLT** | **LHC** | **RHC** | **LFG** | **RFG** | **LPHG** | **RPHG** | **LST** | **RST** | **LMT** | **RMT** | **LIT** | **RIT** |
| --- | --- | --- | --- | --- | --- | --- | --- | --- | --- | --- | --- | --- | --- | --- | --- | --- |
| C1 | 90 | 85 | 95 | 90 | 60 | 60 | 90 | 90 | 90 | 70 | 85 | 90 | 80 | 90 | 85 | 90 |
| C2 | 90 | 85 | 90 | 90 | 55 | 45 | 95 | 85 | 60 | 55 | 80 | 90 | 90 | 80 | 75 | 95 |
| C3 | 100 | 90 | 95 | 85 | 60 | 45 | 95 | 95 | 70 | 60 | 70 | 85 | 80 | 65 | 65 | 80 |
| C4 | 100 | 90 | 90 | 90 | 65 | 45 | 100 | 100 | 55 | 85 | 75 | 65 | 90 | 100 | 100 | 85 |
| C5 | 95 | 90 | 100 | 80 | 65 | 95 | 95 | 100 | 100 | 90 | 80 | 75 | 75 | 85 | 85 | 70 |
| C6 | 55 | 70 | 65 | 80 | 55 | 55 | 40 | 70 | 55 | 50 | 70 | 70 | 60 | 85 | 70 | 85 |
| C7 | 95 | 70 | 100 | 90 | 65 | 50 | 100 | 90 | 70 | 70 | 70 | 85 | 80 | 80 | 90 | 70 |
| C8 | 80 | 65 | 100 | 80 | 50 | 55 | 85 | 100 | 60 | 65 | 65 | 70 | 75 | 80 | 70 | 95 |
| C9 | 100 | 95 | 100 | 100 | 70 | 70 | 100 | 100 | 85 | 80 | 85 | 100 | 95 | 90 | 100 | 95 |
| C10 | 85 | 75 | 80 | 80 | 75 | 75 | 90 | 75 | 70 | 75 | 70 | 75 | 80 | 75 | 75 | 70 |
| C11 | 70 | 90 | 90 | 90 | 70 | 75 | 80 | 80 | 80 | 80 | 70 | 70 | 90 | 90 | 75 | 85 |
| C12 | 95 | 55 | 80 | 70 | 55 | 50 | 100 | 90 | 60 | 60 | 55 | 65 | 75 | 80 | 80 | 95 |
| C13 | 75 | 50 | 70 | 45 | 65 | 60 | 65 | 65 | 40 | 45 | 55 | 45 | 50 | 65 | 55 | 45 |
| C14 | 90 | 80 | 85 | 85 | 65 | 60 | 100 | 100 | 60 | 65 | 70 | 80 | 75 | 75 | 80 | 70 |
| C15 | 100 | 80 | 100 | 100 | 75 | 50 | 95 | 90 | 85 | 65 | 75 | 90 | 80 | 95 | 90 | 80 |
| C16 | 70 | 80 | 65 | 75 | 75 | 55 | 55 | 85 | 65 | 65 | 75 | 80 | 70 | 65 | 55 | 60 |
| C17 | 75 | 75 | 95 | 95 | 55 | 65 | 85 | 95 | 65 | 50 | 65 | 80 | 70 | 95 | 90 | 90 |
| C18 | 100 | 90 | 95 | 90 | 85 | 90 | 100 | 95 | 70 | 85 | 85 | 90 | 90 | 85 | 90 | 90 |
| C19 | 95 | 70 | 100 | 85 | 75 | 75 | 100 | 100 | 80 | 60 | 65 | 90 | 75 | 75 | 75 | 65 |
| C20 | 100 | 75 | 100 | 90 | 65 | 75 | 100 | 90 | 80 | 95 | 75 | 70 | 75 | 90 | 80 | 95 |
| C21 | 85 | 65 | 95 | 80 | 55 | 65 | 95 | 95 | 70 | 65 | 70 | 85 | 65 | 70 | 65 | 90 |
| C22 | 95 | 95 | 95 | 95 | 70 | 60 | 100 | 95 | 65 | 80 | 70 | 75 | 95 | 90 | 90 | 95 |
| LHS1 | 90 | 60 | 70 | 75 | 65 | 40 | 90 | 80 | 70 | 55 | 75 | 60 | 75 | 70 | 45 | 80 |
| LHS2 | 100 | 80 | 100 | 80 | 50 | 65 | 100 | 90 | 75 | 55 | 60 | 85 | 80 | 85 | 85 | 80 |
| LHS3 | 80 | 75 | 80 | 70 | 65 | 55 | 85 | 85 | 70 | 65 | 80 | 75 | 85 | 80 | 75 | 75 |
| LHS4 | 85 | 100 | 95 | 95 | 45 | 50 | 95 | 95 | 60 | 60 | 75 | 85 | 100 | 95 | 85 | 85 |
| LHS5 | 65 | 75 | 95 | 90 | 50 | 60 | 85 | 100 | 55 | 60 | 75 | 80 | 80 | 100 | 60 | 65 |
| LHS6 | 80 | 95 | 85 | 85 | 75 | 55 | 85 | 90 | 55 | 55 | 90 | 65 | 65 | 70 | 80 | 80 |
| LHS7 | 100 | 65 | 100 | 85 | 45 | 60 | 95 | 100 | 50 | 65 | 60 | 55 | 70 | 90 | 60 | 90 |
| LHS8 | 100 | 80 | 85 | 85 | 75 | 50 | 100 | 100 | 70 | 60 | 65 | 75 | 85 | 85 | 85 | 90 |
| LHS9 | 90 | 70 | 85 | 70 | 55 | 55 | 90 | 90 | 55 | 65 | 50 | 65 | 75 | 80 | 75 | 55 |
| LHS10 | 95 | 70 | 90 | 90 | 75 | 50 | 100 | 95 | 80 | 80 | 65 | 60 | 80 | 100 | 80 | 95 |
| LHS11 | 60 | 55 | 70 | 85 | 60 | 60 | 65 | 85 | 40 | 55 | 50 | 75 | 60 | 75 | 55 | 60 |
| LHS12 | 80 | 60 | 85 | 75 | 50 | 45 | 85 | 90 | 55 | 60 | 45 | 65 | 70 | 65 | 70 | 75 |
| LHS13 | 80 | 75 | 75 | 80 | 60 | 55 | 85 | 75 | 60 | 65 | 65 | 70 | 75 | 75 | 70 | 70 |
| LHS14 | 70 | 75 | 90 | 85 | 60 | 55 | 55 | 85 | 55 | 75 | 80 | 80 | 75 | 85 | 65 | 90 |
| LHS15 | 85 | 75 | 95 | 80 | 50 | 40 | 90 | 90 | 55 | 60 | 80 | 60 | 60 | 75 | 75 | 95 |
| LHS16 | 85 | 55 | 95 | 80 | 40 | 40 | 95 | 100 | 55 | 65 | 55 | 60 | 70 | 80 | 50 | 100 |
| LHS17 | 75 | 55 | 75 | 50 | 60 | 60 | 80 | 80 | 65 | 65 | 50 | 55 | 65 | 55 | 65 | 65 |
| LHS18 | 80 | 85 | 90 | 80 | 45 | 75 | 90 | 60 | 70 | 70 | 70 | 60 | 80 | 85 | 75 | 60 |
| RHS1 | 100 | 85 | 100 | 90 | 45 | 60 | 100 | 90 | 65 | 80 | 65 | 70 | 100 | 70 | 95 | 100 |
| RHS2 | 85 | 50 | 80 | 55 | 50 | 50 | 75 | 80 | 40 | 55 | 50 | 50 | 50 | 70 | 55 | 50 |
| RHS3 | 75 | 65 | 85 | 70 | 55 | 55 | 80 | 80 | 60 | 60 | 60 | 50 | 65 | 65 | 70 | 80 |
| RHS4 | 85 | 65 | 85 | 70 | 75 | 60 | 85 | 90 | 60 | 65 | 70 | 55 | 75 | 75 | 75 | 65 |
| RHS5 | 85 | 65 | 80 | 75 | 65 | 70 | 90 | 85 | 60 | 65 | 65 | 75 | 60 | 80 | 70 | 75 |
| RHS6 | 90 | 70 | 65 | 65 | 75 | 55 | 95 | 70 | 75 | 65 | 70 | 65 | 75 | 70 | 85 | 90 |
| RHS7 | 85 | 70 | 85 | 85 | 75 | 35 | 85 | 90 | 65 | 65 | 55 | 85 | 70 | 80 | 55 | 80 |
| RHS8 | 65 | 65 | 70 | 45 | 70 | 55 | 65 | 80 | 50 | 60 | 65 | 55 | 55 | 40 | 50 | 50 |
| RHS9 | 95 | 75 | 90 | 80 | 55 | 50 | 95 | 90 | 70 | 65 | 65 | 65 | 70 | 85 | 80 | 80 |
| RHS10 | 90 | 65 | 95 | 60 | 70 | 50 | 95 | 95 | 45 | 50 | 65 | 50 | 80 | 75 | 75 | 70 |
| RHS11 | 70 | 65 | 60 | 60 | 55 | 60 | 70 | 85 | 60 | 60 | 60 | 55 | 60 | 75 | 60 | 70 |
| RHS12 | 65 | 60 | 65 | 75 | 45 | 65 | 70 | 75 | 70 | 50 | 55 | 70 | 65 | 65 | 70 | 70 |
| RHS13 | 95 | 95 | 95 | 80 | 80 | 80 | 95 | 100 | 75 | 75 | 90 | 85 | 90 | 90 | 90 | 80 |
| RHS14 | 90 | 90 | 100 | 90 | 55 | 60 | 90 | 95 | 65 | 70 | 90 | 90 | 85 | 75 | 85 | 80 |
| RHS15 | 75 | 60 | 65 | 70 | 55 | 70 | 80 | 60 | 75 | 60 | 65 | 75 | 60 | 65 | 60 | 75 |
| RHS16 | 80 | 85 | 90 | 90 | 65 | 70 | 85 | 90 | 50 | 55 | 75 | 85 | 80 | 80 | 85 | 90 |
| RHS17 | 65 | 75 | 70 | 55 | 60 | 50 | 70 | 75 | 65 | 55 | 55 | 60 | 60 | 60 | 65 | 65 |
| RHS18 | 95 | 85 | 90 | 75 | 65 | 70 | 95 | 95 | 70 | 60 | 80 | 70 | 80 | 80 | 90 | 70 |
| RHS19 | 85 | 85 | 85 | 70 | 80 | 70 | 90 | 95 | 55 | 55 | 70 | 50 | 95 | 75 | 80 | 80 |
| RHS20 | 70 | 60 | 45 | 50 | 50 | 50 | 80 | 70 | 60 | 40 | 60 | 60 | 55 | 45 | 75 | 75 |
| RHS21 | 95 | 85 | 90 | 85 | 50 | 40 | 90 | 90 | 50 | 50 | 70 | 75 | 90 | 80 | 95 | 90 |
| RHS22 | 65 | 70 | 85 | 85 | 50 | 65 | 85 | 95 | 50 | 65 | 65 | 80 | 85 | 70 | 65 | 90 |
| RHS23 | 95 | 85 | 90 | 85 | 65 | 45 | 95 | 85 | 55 | 60 | 55 | 80 | 75 | 80 | 100 | 95 |

**Table S4:** Average classification accuracies of all folds in different ROIs in Controls, LHS and RHS individuals in the words vs rest experiment**.**

| **Subjects** | **LmesT** | **LLT** | **RmesT** | **RLT** | **LHC** | **RHC** | **LFG** | **RFG** | **LPHG** | **RPHG** | **LST** | **RST** | **LMT** | **RMT** | **LIT** | **RIT** |
| --- | --- | --- | --- | --- | --- | --- | --- | --- | --- | --- | --- | --- | --- | --- | --- | --- |
| C1 | 85 | 85 | 85 | 85 | 45 | 30 | 85 | 85 | 50 | 55 | 90 | 80 | 85 | 80 | 85 | 80 |
| C2 | 90 | 80 | 80 | 60 | 45 | 55 | 90 | 75 | 70 | 45 | 75 | 55 | 80 | 60 | 80 | 70 |
| C3 | 95 | 90 | 95 | 95 | 60 | 50 | 95 | 95 | 70 | 60 | 95 | 80 | 85 | 80 | 80 | 85 |
| C4 | 100 | 100 | 85 | 75 | 50 | 45 | 100 | 75 | 65 | 65 | 85 | 75 | 100 | 85 | 95 | 75 |
| C5 | 85 | 90 | 80 | 85 | 55 | 60 | 85 | 80 | 75 | 75 | 90 | 95 | 80 | 85 | 100 | 75 |
| C6 | 60 | 65 | 55 | 70 | 60 | 40 | 80 | 60 | 65 | 55 | 65 | 65 | 70 | 75 | 55 | 80 |
| C7 | 75 | 65 | 60 | 70 | 55 | 50 | 75 | 60 | 50 | 65 | 65 | 80 | 70 | 55 | 70 | 65 |
| C8 | 80 | 60 | 60 | 65 | 30 | 40 | 85 | 80 | 55 | 50 | 70 | 55 | 65 | 50 | 65 | 65 |
| C9 | 90 | 90 | 95 | 90 | 65 | 75 | 90 | 95 | 80 | 80 | 70 | 85 | 85 | 70 | 85 | 80 |
| C10 | 85 | 90 | 85 | 90 | 60 | 80 | 85 | 85 | 80 | 85 | 85 | 95 | 85 | 80 | 85 | 90 |
| C11 | 70 | 65 | 60 | 60 | 70 | 50 | 75 | 65 | 65 | 55 | 70 | 55 | 65 | 60 | 75 | 45 |
| C12 | 100 | 70 | 75 | 65 | 50 | 40 | 100 | 85 | 50 | 40 | 60 | 45 | 85 | 60 | 85 | 70 |
| C13 | 60 | 60 | 60 | 60 | 35 | 40 | 55 | 75 | 60 | 45 | 60 | 50 | 50 | 65 | 55 | 75 |
| C14 | 85 | 90 | 70 | 85 | 60 | 60 | 80 | 75 | 60 | 50 | 80 | 85 | 90 | 75 | 80 | 70 |
| C15 | 100 | 90 | 100 | 90 | 55 | 50 | 95 | 100 | 75 | 75 | 90 | 85 | 85 | 90 | 85 | 95 |
| C16 | 65 | 90 | 65 | 70 | 55 | 65 | 60 | 70 | 60 | 60 | 90 | 75 | 80 | 80 | 70 | 75 |
| C17 | 85 | 75 | 60 | 70 | 50 | 50 | 85 | 85 | 60 | 40 | 75 | 65 | 85 | 80 | 90 | 70 |
| C18 | 100 | 90 | 75 | 85 | 70 | 60 | 100 | 75 | 75 | 70 | 85 | 85 | 90 | 85 | 85 | 75 |
| C19 | 90 | 65 | 90 | 80 | 40 | 40 | 100 | 85 | 55 | 50 | 65 | 80 | 65 | 55 | 65 | 65 |
| C20 | 85 | 85 | 80 | 75 | 45 | 40 | 85 | 100 | 60 | 75 | 85 | 65 | 80 | 70 | 85 | 70 |
| C21 | 60 | 70 | 90 | 70 | 55 | 50 | 70 | 90 | 60 | 55 | 90 | 75 | 65 | 65 | 65 | 85 |
| C22 | 80 | 90 | 80 | 90 | 45 | 65 | 85 | 85 | 60 | 90 | 80 | 95 | 100 | 95 | 100 | 90 |
| LHS1 | 90 | 85 | 70 | 75 | 55 | 45 | 90 | 75 | 70 | 65 | 80 | 60 | 70 | 80 | 75 | 70 |
| LHS2 | 85 | 80 | 85 | 80 | 50 | 45 | 85 | 85 | 55 | 65 | 65 | 85 | 65 | 70 | 75 | 90 |
| LHS3 | 95 | 90 | 85 | 90 | 80 | 70 | 90 | 90 | 80 | 75 | 80 | 80 | 90 | 85 | 80 | 85 |
| LHS4 | 80 | 95 | 70 | 80 | 55 | 50 | 80 | 90 | 45 | 50 | 85 | 75 | 85 | 50 | 95 | 60 |
| LHS5 | 80 | 70 | 80 | 85 | 40 | 50 | 80 | 95 | 55 | 50 | 70 | 85 | 75 | 90 | 75 | 70 |
| LHS6 | 75 | 100 | 80 | 85 | 70 | 55 | 80 | 75 | 65 | 50 | 100 | 65 | 75 | 85 | 85 | 80 |
| LHS7 | 95 | 85 | 90 | 75 | 80 | 55 | 90 | 85 | 65 | 70 | 65 | 80 | 95 | 65 | 75 | 70 |
| LHS8 | 100 | 80 | 90 | 90 | 65 | 60 | 100 | 90 | 75 | 55 | 70 | 75 | 95 | 80 | 90 | 100 |
| LHS9 | 85 | 85 | 75 | 75 | 60 | 45 | 95 | 85 | 70 | 70 | 75 | 65 | 80 | 50 | 90 | 80 |
| LHS10 | 95 | 70 | 100 | 80 | 50 | 50 | 95 | 95 | 55 | 60 | 65 | 75 | 75 | 90 | 65 | 100 |
| LHS11 | 65 | 40 | 55 | 35 | 55 | 35 | 80 | 65 | 35 | 35 | 45 | 45 | 50 | 50 | 80 | 35 |
| LHS12 | 75 | 75 | 65 | 65 | 50 | 45 | 80 | 70 | 50 | 60 | 60 | 55 | 80 | 50 | 70 | 65 |
| LHS13 | 85 | 90 | 75 | 60 | 45 | 60 | 95 | 85 | 60 | 60 | 75 | 70 | 65 | 55 | 75 | 65 |
| LHS14 | 60 | 70 | 50 | 70 | 50 | 40 | 60 | 60 | 40 | 45 | 70 | 60 | 80 | 65 | 60 | 65 |
| LHS15 | 95 | 95 | 70 | 60 | 45 | 45 | 95 | 85 | 45 | 45 | 85 | 75 | 65 | 65 | 95 | 75 |
| LHS16 | 90 | 75 | 85 | 85 | 55 | 55 | 95 | 100 | 50 | 80 | 60 | 65 | 85 | 80 | 95 | 95 |
| LHS17 | 70 | 55 | 60 | 55 | 55 | 30 | 80 | 60 | 60 | 60 | 50 | 50 | 65 | 60 | 55 | 70 |
| LHS18 | 95 | 60 | 60 | 65 | 50 | 35 | 95 | 75 | 60 | 50 | 65 | 65 | 65 | 70 | 90 | 70 |
| RHS1 | 95 | 85 | 95 | 65 | 50 | 40 | 95 | 95 | 75 | 60 | 65 | 60 | 90 | 60 | 90 | 65 |
| RHS2 | 70 | 75 | 45 | 55 | 20 | 55 | 70 | 65 | 40 | 40 | 55 | 50 | 60 | 45 | 65 | 65 |
| RHS3 | 80 | 85 | 75 | 65 | 70 | 65 | 90 | 75 | 65 | 55 | 60 | 80 | 80 | 65 | 85 | 70 |
| RHS4 | 55 | 65 | 55 | 55 | 45 | 25 | 70 | 60 | 35 | 45 | 65 | 55 | 50 | 55 | 60 | 40 |
| RHS5 | 80 | 70 | 75 | 85 | 55 | 55 | 80 | 70 | 55 | 75 | 70 | 80 | 60 | 85 | 65 | 65 |
| RHS6 | 85 | 70 | 70 | 60 | 65 | 60 | 85 | 55 | 80 | 65 | 80 | 60 | 60 | 55 | 70 | 60 |
| RHS7 | 75 | 85 | 85 | 90 | 60 | 55 | 85 | 85 | 70 | 65 | 95 | 85 | 75 | 90 | 55 | 85 |
| RHS8 | 65 | 45 | 60 | 45 | 50 | 65 | 75 | 50 | 70 | 45 | 50 | 40 | 50 | 45 | 50 | 50 |
| RHS9 | 80 | 70 | 70 | 65 | 35 | 60 | 80 | 70 | 55 | 35 | 60 | 50 | 70 | 80 | 80 | 65 |
| RHS10 | 95 | 75 | 75 | 65 | 65 | 85 | 90 | 80 | 45 | 45 | 65 | 55 | 60 | 70 | 65 | 70 |
| RHS11 | 85 | 75 | 70 | 75 | 65 | 55 | 85 | 55 | 75 | 70 | 55 | 55 | 55 | 80 | 70 | 65 |
| RHS12 | 45 | 65 | 55 | 60 | 55 | 60 | 60 | 60 | 45 | 45 | 65 | 70 | 65 | 65 | 70 | 50 |
| RHS13 | 95 | 95 | 80 | 85 | 50 | 50 | 95 | 90 | 70 | 55 | 95 | 80 | 100 | 85 | 95 | 90 |
| RHS14 | 75 | 40 | 60 | 40 | 30 | 40 | 95 | 90 | 40 | 50 | 40 | 40 | 40 | 55 | 50 | 35 |
| RHS15 | 90 | 60 | 60 | 70 | 55 | 60 | 90 | 70 | 55 | 60 | 55 | 65 | 60 | 80 | 85 | 65 |
| RHS16 | 70 | 85 | 70 | 85 | 55 | 75 | 95 | 75 | 50 | 55 | 85 | 80 | 80 | 85 | 95 | 80 |
| RHS17 | 75 | 60 | 55 | 70 | 45 | 50 | 75 | 75 | 50 | 45 | 70 | 60 | 50 | 75 | 75 | 50 |
| RHS18 | 100 | 95 | 95 | 85 | 45 | 65 | 100 | 100 | 70 | 55 | 85 | 90 | 85 | 65 | 95 | 65 |
| RHS19 | 75 | 50 | 70 | 45 | 50 | 40 | 80 | 85 | 40 | 55 | 50 | 30 | 60 | 50 | 40 | 55 |
| RHS20 | 50 | 45 | 35 | 40 | 35 | 45 | 45 | 45 | 45 | 60 | 30 | 35 | 45 | 50 | 45 | 50 |
| RHS21 | 90 | 100 | 60 | 70 | 55 | 40 | 90 | 65 | 45 | 60 | 100 | 60 | 85 | 80 | 90 | 80 |
| RHS22 | 80 | 75 | 75 | 75 | 60 | 65 | 80 | 90 | 50 | 75 | 70 | 70 | 85 | 75 | 55 | 85 |
| RHS23 | 90 | 80 | 75 | 80 | 70 | 50 | 100 | 90 | 65 | 60 | 75 | 90 | 85 | 75 | 95 | 85 |
